# Embolization of Patent Foramen Ovale Closure Device—Rare Complication and Unique Management Approach

**DOI:** 10.3390/medicina60050717

**Published:** 2024-04-26

**Authors:** Mila Kovacevic, Marko Atanaskovic, Katarina Obradovic, Mirko Todic, Branislav Crnomarkovic, Marija Bjelobrk, Snezana Bjelic, Milenko Cankovic, Aleksandra Milovancev, Ilija Srdanovic

**Affiliations:** 1 Faculty of Medicine, University of Novi Sad, 21000 Novi Sad, Serbia; atanaskovic.marko5@gmail.com (M.A.); obradovic216@gmail.com (K.O.); mirko.todic@mf.uns.ac.rs (M.T.); branislavc.89@gmail.com (B.C.); marija.bjelobrk@mf.uns.ac.rs (M.B.); milenko.cankovic@gmail.com (M.C.); aleksandra.milovancev@mf.uns.ac.rs (A.M.); ilija.srdanovic@mf.uns.ac.rs (I.S.); 2 Institute of Cardiovascular Diseases of Vojvodina, 21204 Sremska Kamenica, Serbia; snezana.bjelic@mf.uns.ac.rs

**Keywords:** patent foramen ovale, closure device, embolization, complication, interventional management

## Abstract

Percutaneous closure of the patent foramen ovale (PFO) is generally regarded as a safe and effective procedure, indicated in patients with a prior PFO-associated stroke. While it is highly safe, rarely, it could be accompanied by a migration of the device, mainly caused by the interplay of a specific PFO morphology and inappropriate device sizing. Herein, we outline a seldom-observed complication of an unintentional detachment of the PFO closure device during implantation, leading to its migration into the abdominal aorta, and a unique management approach. Due to the inability to recapture the occluder with a snare, which is considered to be a mainstay of endovascular retrieval methods, two coronary guidewires were maneuvered through the mesh of the occluder and then captured with a snare proximally to the occluder. This innovative dual-wire–snare system was carefully pulled to the common femoral artery, a position deemed suitable for surgical extraction via arteriotomy, which was achieved successfully.

## 1. Introduction

A patent foramen ovale (PFO) is a relatively prevalent cardiac anomaly, found in approximately 25–30% of the general population, and it is regarded as the cause of 50% of cryptogenic strokes, especially in younger individuals [1,2]. The current recommendation of the European Society of Cardiology for the treatment of a PFO in patients with a paradoxical embolism who are 18–60 years old and with all the other causes of cryptogenic stroke excluded is a percutaneous transcatheter PFO closure along with antiplatelet therapy [3]. Although percutaneous PFO closure is considered as a safe and effective procedure, this intervention carries a certain risk of device embolization. Herein, we present a case of PFO closure device embolization to the abdominal aorta, which was successfully managed by percutaneous endovascular retrieval followed by surgical extraction.

## 2. Case Presentation

**Figure 1:**
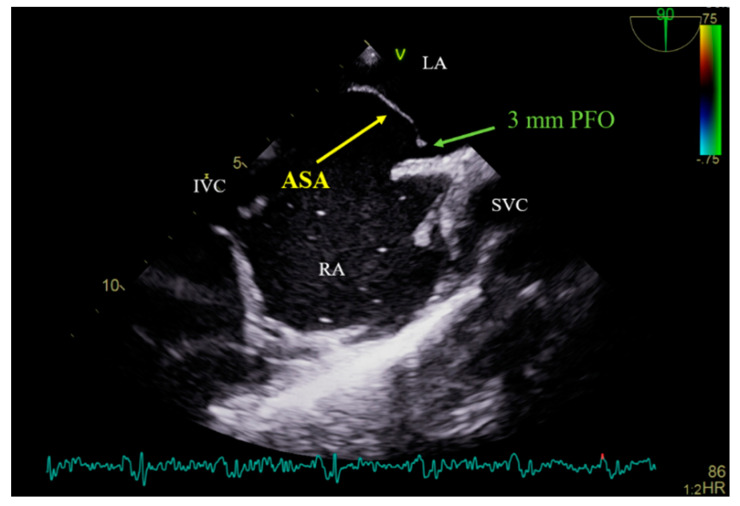
Bicaval view TEE showing PFO with an atrial septal aneurysm. TEE—transesophageal echocardiography; PFO—patent foramen ovale; ASA—atrial septal aneurysm; LA—left atrium; RA—right atrium; IVC—inferior vena cava; SVC—superior vena cava.

A 54-year-old female patient who suffered an ischemic stroke with transient right-hand paresis and motor dysphasia was admitted for a scheduled percutaneous transcatheter closure of the patent foramen ovale (PFO). Prior investigations, including the magnetic resonance imaging of the head and neck, 24 h Holter monitoring for arrhythmia, and blood testing for a hypercoagulable state, were performed and yielded unremarkable results. Subsequent diagnostic tests, including a transcranial Doppler (TCD) bubble test and saline contrast transthoracic echocardiography (TTE), revealed a right-to-left shunt at the level of the interatrial septum (IAS). This was confirmed by transesophageal echocardiography (TEE), which identified the presence of the PFO with an atrial septal aneurysm (ASA) and an abnormal septal excursion (Figure 1). Based on a thorough investigation, all the other potential causes of a cryptogenic stroke, such as major vessel atherosclerosis, small vessel disease, atrial fibrillation with cardioembolism, artery dissection, a hypercoagulable state, uncontrolled hypertension or diabetes mellitus, and autoimmune disease, were excluded. The diagnosis of a PFO-related stroke was established. Therefore, the percutaneous endovascular closure of the PFO was the management strategy of choice.

**Figure 2:**
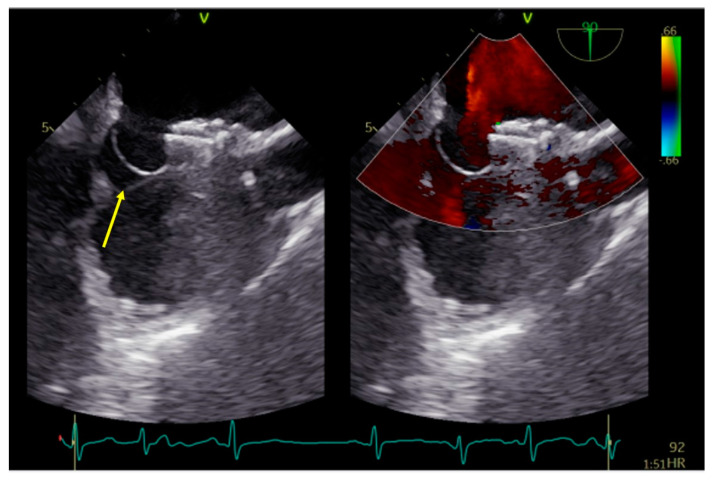
PFO occluder on IAS depicting a large portion of aneurysmatic and not stabilized septum (arrow). PFO−patent foramen ovale; IAS−interatrial septum.

The procedure began with an ultrasound-guided puncture of the right femoral vein. A MACH 1™ Multipurpose MP1 catheter (Boston Scientific, Marlborough, MA, USA) was navigated through the PFO over the 0.035″J (Merit Medical Systems Inc., South Jordan, UT, USA) guidewire and accurately positioned in the upper left pulmonary vein. Subsequently, a 9 French (F) delivery system was placed over the Amplatz wire. Due to the aneurysmatic septum, the largest available PFO closure device, Figulla^®^ Flex II 27/30 mm PFO Occluder (Occlutech^®^, Jena, Germany), was selected, and sequential deployment of the left atrial and right atrial disks was completed. However, a significant portion of the aneurysmatic and floppy IAS towards the inferior vena cava remained unstabilized (Figure 2).

**Figure 3:**
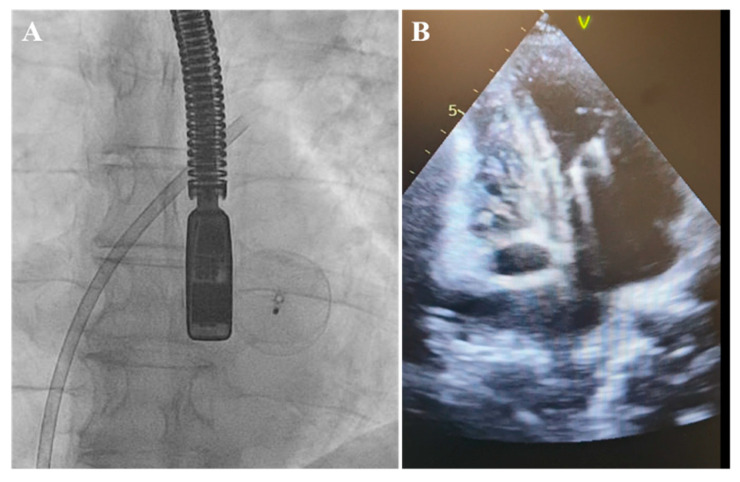
Detached occluder in the projection of the left ventricle. (**A**) Fluoroscopy. (**B**) Transthoracic echocardiography.

Therefore, in response to the notably aneurysmatic, floppy, and thin interatrial septum, to prevent the unintended slippage of the PFO closure device, we undertook efforts to reposition the occluder to stabilize a large portion of the septum despite recognizing that this might have necessitated the use of a larger device. Following this adjustment, the PFO occluder was unexpectedly and unintentionally detached, without the release of the device safety mechanism. Initially, the position of the PFO occluder was confirmed on the radiogram in the projection of the left atrium (LA) and then in the left ventricle (LV) projection (Figure 3).

**Figure 4:**
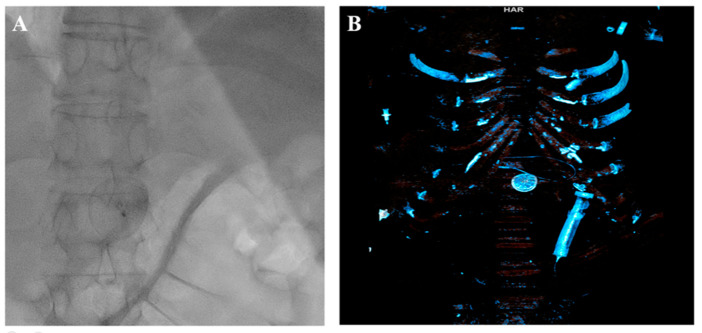
Occluder in the abdominal aorta. (**A**) Fluoroscopy. (**B**) Computed tomography.

The attempts to snare it from the LA and LV were unsuccessful. Furthermore, the device promptly migrated to the abdominal aorta, located below the diaphragm at the junction of the truncus coeliacus, which was confirmed via a CT scan (Figure 4).

At that point, to circumvent the necessity for surgery, which entails a laparotomy involving retroperitoneal access and multi-site aortic clamping, thereby increasing the retrieval complexity, the duration of the procedure, and the risk of complications, our objective was either complete retrieval of the occluder using a snare or positioning the occluder to the common femoral artery, making it suitable for surgical extraction. 

**Figure 5:**
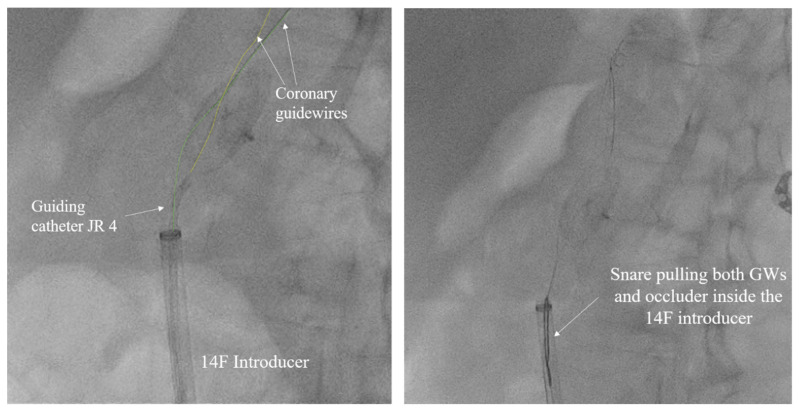
Amplatz Goose Neck snare complemented with two coronary guidewires (GWs) in a unique system for occluder retrieval.

The left common femoral artery (CFA) was promptly punctured, followed by the placement of a 14F introducer. Several attempts were undertaken to recapture the right atrial disc button of the PFO occluder using a 15 mm Amplatz Goose Neck snare (Medtronic, Minneapolis, MN, USA), but these efforts were unsuccessful. The position of the device, trapped within the aorta, with the right atrial disk button opposite to the aortic wall, disabled access to the device even with a 6 F guiding catheter Judkins right (JR) 4 used to navigate and reach the occluder button. This step was succeeded by the introduction of two coronary guidewires with higher tip loads, namely the Pilot 150 (Abbott Vascular, Santa Clara, CA, USA) and Samurai RC (Boston Scientific, Marlborough, MA, USA), one through the JR 4 guiding catheter already positioned and the second one directly through the 14F introducer. These wires were subsequently maneuvered, one by one, through the mesh of the PFO occluder (Figure 5). The JR 4 guiding catheter was withdrawn, and a 15 mm Amplatz Goose Neck snare was introduced through the 14F introducer. Following this, two coronary guidewires were captured proximally to the PFO occluder using a 15 mm Amplatz Goose Neck snare and subsequently pulled, facilitating the partial retraction of the PFO occluder into the 14F introducer (Figure 5, Video S1).

**Figure 6:**
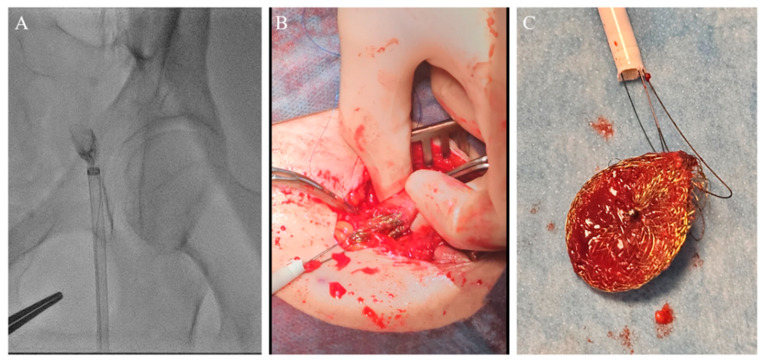
(**A**) Occluder within 14F introducer at the level of the left common femoral artery (CFA). (**B**) Surgical extraction via arteriotomy of CFA. (**C**) Extracted occluder wrapped with two coronary guidewires.

The entire wire–occluder system within the 14F introducer was then carefully retracted to the common femoral artery to a position deemed suitable for surgical extraction via arteriotomy, which was achieved successfully (Figure 6). 

A control TEE after the procedure showed that no damage to the interatrial septum occurred during the procedure. Furthermore, after the retrieval of the occluder and close inspection of the ball-forceps connection of the device, we could not detect any technical malfunction. The patient was discharged on the fourth day with the recommendation to continue lifelong antiplatelet therapy with acetylsalicylic acid.

## 3. Discussion

The migration of a PFO closure device is a rare but serious complication, occurring in no more than 0.3–0.7% of cases, as reported by the current literature [1,2,3]. As of now, the percutaneous recapture of displaced intravascular objects is preferred over surgical procedures. As the embolization of a PFO or an atrial septal defect (ASD) closure device is an uncommon complication of a percutaneous transcatheter PFO/ASD closure, the studies detailing the causes, clinical features, prevention, and management of this complication are sparse.

The potential causes of closure device migration include the incorrect sizing of the device, inaccurate deployment, atrial septum tearing due to device manipulation, or inadequate operator experience [4]. Additionally, other documented risk factors for device dislodgement and subsequent migration include an ASA, a hypermobile atrial septum (septal excursion ≥10 mm), a thick septum secundum (>10 mm), a large Eustachian valve, or a lengthy tunnel [4,5].

Moreover, in all the cases involving an ASA, there is a higher likelihood of underestimating the required device size. Even though TEE is generally the most sensitive method for device sizing, in some instances, balloon sizing to determine not just the size but the shape of a PFO can be conducted [4]. However, in patients with an aneurysmatically altered septum, it can be accompanied by a higher risk of iatrogenic septal rupture. Furthermore, in the case reported by Goel et al. [4], despite performing balloon defect sizing, there was an underestimation regarding the sizing. Therefore, to adequately encompass the entire redundant septum, the use of a larger device is recommended. This approach is designed to prevent the device from slipping off the septum secundum and to permit the mobility of the septum primum between the left and right atrial disks. However, achieving this is challenging as the device may impinge on nearby structures, such as the aortic root or the mitral and tricuspid valves. 

In our patient’s case, although we anticipated all the features that may impact the proper deployment of the PFO occluder, such as the presence of the ASA and hypermobile atrial septum, and by choosing the largest available PFO device, we could not foresee the unintentional detachment of the device, caused either by the inadequate loading of the device, device manipulation, or both. 

Moreover, the presence of an ASA caused additional manipulation during the device positioning, generating an extra force on the ball-forceps connection of the device, which, in conjunction with inadequate loading, might cause the unintentional detachment of the occluder, representing one of the contributing factors to the complication encountered. Other predisposing factors for device migration, such as a long tunnel, a prominent Eustachian valve, and a thick septum secundum, were beyond the present focus.

The abdominal aorta is one of the many locations where the device can dislodge, and the rest are the cardiac chambers, pulmonary artery, and all the parts of the aorta up to the iliac arteries [6,7].

Although the embolization of a closure device to the left ventricular outflow tract and the aorta is exceedingly rare, it can be life-threatening. As such, prompt and appropriate management is imperative. In the study by Levi et al. [7], 70% of the patients with migrated ASD closure devices underwent percutaneous endovascular retrieval, while the remainder required surgical intervention. Other studies have indicated that surgical retrieval was necessary in 77% of the cases, with percutaneous retrieval being feasible in only 17% [6,8]. The decision regarding the appropriate course of action, percutaneous or surgical, should be based on multiple factors, including the location of the migration, the placement-to-embolization interval, the degree of device endothelialization, the size of the device relative to the aortic radius, the availability of specific instruments, and the patient’s characteristics and condition. 

As device endothelialization is a long-term process, in our case, considering the periprocedural immediate embolization, endothelialization was not a significant concern. Therefore, surgical retrieval was not mandatory. Furthermore, given the location of the embolized device in the abdominal aorta below the diaphragm, the surgical extraction of the device would require an approach to the retroperitoneal space, making surgery extremely difficult and hazardous. Moreover, considering the relatively good patient condition and available access and instruments, an endovascular approach was reasonable and justified. 

In general, devices that embolize to the atria should be pulled into the inferior vena cava below the hepatic veins, while devices that embolize to the ventricles should be safely pushed forward across the semilunar valves to the aorta or pulmonary artery to avoid the possibility of entangling the device into the mitral or tricuspid valve [7]. Once the device is in a safe position, different percutaneous retrieval techniques could be applied [4,5,6,9,10].

Principally, the backbone of all the techniques was snaring the device by capturing the screw mechanism or a button of the right atrial disk and the retrieval of the device into the large bore sheath, usually at least 2 F larger than the recommended delivery sheath [7]. Other authors, however, strongly recommend a 24 F sheath, when feasible, to minimize the tension required for device retrieval into the sheath, thus preventing the snare from loosening its grip on the occluder. It is particularly essential when the placement-to-embolization interval is longer, creating a higher probability of stripping the thrombus or adhered tissue from the occluder during the retrieval [6]. In some case reports, the snaring system was supplemented either with an additional snare [10], endobronchial forceps [6], or a multipurpose biopsy forceps [9]. Although bioptomes were ineffective in grabbing the screw or button mechanism of the device, they were used as adjunctive tools either to maneuver the devices into a suitable position for snaring or to stabilize the occluder, ensuring successful snaring. However, in a case reported by Musuraca G et al., involving late PFO occluder dislodgment into the abdominal aorta across both common iliac arteries, snaring the occluder proved to be unsuccessful in retrieving it into a 20 French sheath due to the device’s lack of compressibility over time. The occluder was pulled into the left common iliac artery and subsequently surgically extracted via a left-sided Rutherford Morrison incision [11].

In our case, we employed a unique system consisting of a 15 mm Amplatz Goose Neck snare, which was complemented with a dual-wire unit featuring Pilot 150 and Samurai RC coronary wires. These components were integrated into a sophisticated and efficient snaring technique, which was eventually followed by a CFA arteriotomy.

## 4. Conclusions

To the best of our knowledge, this report presents the first documented case of percutaneous endovascular retrieval that combines a dual-wire system with a Goose Neck snare and a CFA arteriotomy in the successful management of the periprocedural embolization of a PFO occluder.

This case underscores the importance of imaging in the early detection of predisposing factors for the embolization of the closure device, as well as the importance of careful inspection during the loading of the device and careful manipulation during deployment. Ultimately, the timely percutaneous management of device migration, utilizing appropriate snaring techniques, is paramount.

## Data Availability

Data are contained within the article.

## Supplementary Materials

The following supporting information can be downloaded at: https://www.mdpi.com/article/10.3390/medicina60050717/s1, Video S1: PFO occluder retrieval with the dual coronary guidewire and snare system.
